# Recurrence of depression in the perinatal period: Clinical features and associated vulnerability markers in an observational cohort

**DOI:** 10.1371/journal.pone.0212964

**Published:** 2019-02-22

**Authors:** Nina M. Molenaar, Marlies E. Brouwer, Astrid M. Kamperman, Huibert Burger, Alishia D. Williams, Witte J. G. Hoogendijk, Claudi L. H. Bockting, Mijke P. Lambregtse-van den Berg

**Affiliations:** 1 The Department of Psychiatry, Erasmus Medical Center, Rotterdam, The Netherlands; 2 The Department of Psychiatry, Amsterdam University Medical Centers, location AMC, University of Amsterdam, Amsterdam, The Netherlands; 3 The Department of Clinical Psychology, Utrecht University, Utrecht, The Netherlands; 4 The Department of General Practice and Elderly Care Medicine, University Medical Center Groningen, University of Groningen, Groningen, The Netherlands; 5 Faculty of Science, School of Psychology, The University of New South Wales, Sydney, Australia; 6 The Department of Child and Adolescent Psychiatry, Sophia Children’s Hospital, Erasmus Medical Center, Rotterdam, The Netherlands; St. Paul's Hospital Millenium Medical College, ETHIOPIA

## Abstract

**Objective:**

Antidepressant medication is commonly used for the prevention of depression recurrence in the perinatal period, yet it is unknown what vulnerability markers may play a role in recurrence. The objective of the current study was to provide a descriptive overview of the associated characteristics of women who experienced a perinatal recurrence of depression despite ongoing antidepressant use, and further, to identify clinically measurable vulnerability markers associated with recurrence.

**Methods:**

Eighty-five pregnant women with a history of depression who used antidepressants (e.g. Selective Serotonin Reuptake Inhibitors or Serotonin and Noradrenaline Reuptake Inhibitors) at the start of the study were included. Clinical features, including information on psychiatric history and antidepressant use, were collected throughout the perinatal period (in this study defined as the period between 12 weeks of pregnancy untill three months postpartum). The clinical features of women experiencing recurrence of depression were described in detail. To identify vulnerability markers associated with recurrence of depression, we performed exploratory univariable logistic regression analyses.

**Results:**

Eight women (9.4%) experienced a recurrence of depression; two during pregnancy and six in the first 12 weeks postpartum. All women with recurrence of depression had first onset of depression during childhood or adolescence and had at least 2 psychiatric co-morbidities. Identification of vulnerability markers associated with recurrence of depression yielded associations with depressive symptoms around 16 weeks of pregnancy (OR 1.28, 95%CI 1.08–1.52), number of psychiatric co-morbidities (OR 1.89, 95%CI 1.16–3.09) and duration of antidepressant use (OR 1.01, 95%CI 1.00–1.02).

**Conclusion:**

Implementing adequate risk assessment in pregnant women who use antidepressants can help identify predictors for recurrence of depression in future studies and thus ultimately lead to improved care.

## Introduction

Mental illness during the perinatal period (i.e. during pregnancy up to three months postpartum) is a common health problem [1], with approximately 25% of women experiencing any psychiatric disorder in this period [2]. Perinatal depressive disorder is most common, with a recent meta-analysis observing a pooled prevalence of 11.9% [3]. Untreated perinatal depression is not only unfavourable for the mother; it is also associated with adverse outcomes in the offspring [4]. Exposure to antenatal depressive disorder is associated with increased risks of premature delivery, low birth weight [5–7], and behavioural, emotional, cognitive and motor problems in early childhood [8–10]. Ante- and postnatal depression can furthermore influence the mother-infant relationship, posing increased risks for poor infant development [11–13].

Prevention or treatment of perinatal depression is therefore of importance. Several treatment options are available [14], but international guidelines differ in their recommendations [15] and clinicians are frequently noncompliant [16]. Antidepressant medication is an increasingly used treatment option, either for prevention of recurrence of depression or as acute treatment in newly depressed patients [17–19]. Perinatal prescription rates of antidepressants vary per study setting and range from 2.1% to 13.4% [17, 19–21].

The preventive effect of continued antidepressant use in recovered women during the perinatal period remains unclear. A systematic review assessing the effectiveness of antidepressants for prevention of postnatal depression, based on observational studies, could not draw any clear conclusions due to low statistical power [22]. Two studies with a prospective naturalistic design followed women who continued or tapered antidepressants, from their first trimester throughout their pregnancy [23, 24]. One study showed an increased risk of recurrence in women who discontinued their medication compared to women who continued their medication (68% vs. 26%) [23], the other study observed similar recurrence rates in women continuing or discontinuing antidepressants (16% in total) [24]. A large retrospective administrative data study comparing women who continued antidepressants during pregnancy to those who discontinued showed women who continued were twice as likely (OR 2.0, 95%CI 1.8–2.2) to have a depression inpatient stay [25].

From a clinical perspective, recognizing which pregnant women using antidepressants are at risk for recurrence is vital. With this knowledge, clinicians could more accurately identify and inform patients, and subsequently arrange additional guidance when necessary. Collectively these efforts could help promote the use of individualized patient-centred care, and potentially prevent negative effects in the offspring. The purpose of the current study was to describe cases with perinatal recurrence of depression out of a group of pregnant women using antidepressants in their first trimester in detail. Clinical features of the women with recurrence were inspected and reported. Additionally, vulnerability markers associated with recurrence that are easily collected during routine care, were explored.

## Methods

### Setting and population

The present study is an observational study of 85 pregnant women that used antidepressants at the start of the study and had a history of depression. This study was part of a larger nation-wide research project on antidepressants, including both a randomized controlled trial (RCT), called ‘Stop or Go’, in which women are randomized to continue or discontinue antidepressants during pregnancy [26] and an observational cohort. The present manuscript does not report on women from the RCT. The Medical Ethical Committee of the Erasmus Medical Center approved both the RCT and observational cohort (MEC-2014-505).

Women were recruited for both studies during their prenatal booking visit in midwifery practices and hospitals, through general practitioners, or through advertisement in (social) media. When a potential eligible woman was identified, study researchers gave counselling about the RCT and observational cohort. When women were unwilling or not eligible to participate in the RCT, they were counseled for the observational cohort. Written informed consent was necessary for participation.

For the present observational study, participants were considered eligible if they 1) were between 12 and 16 weeks pregnant, 2) used a Selective Serotonin Reuptake Inhibitor (SSRI), Selective Serotonin and Noradrenalin Reuptake Inhibitor (SNRI) or Tricyclic Antidepressant (TCA), 3) had a history of at least one depressive episode as assessed by the Structured Clinical Interview for DSM-IV Axis I Disorders (SCID-I) [27], and 4) did not have a current diagnosis of depression according to the SCID-I. Women without sufficient proficiency in Dutch or English were excluded. No intervention was given in the observational cohort and participants were free to decide themselves whether to continue or taper their antidepressants during study follow-up. Participants were recruited between April 2015 and February 2018.

### Perinatal recurrence of depression

We assessed relapse and recurrence, as defined by the SCID-I, during pregnancy and up to 12 weeks postpartum. Relapse is defined as the re-emergence of depressive symptoms during the remission phase (being symptom-free from illness), but before full recovery (the absence of symptoms for at least 4 months following the onset of remission). Recurrence is defined as the onset of a new depressive episode during the recovery phase or long remission phase. We use the word recurrence for further reference to both relapse and recurrence. The SCID-I was assessed before 16 weeks of pregnancy (baseline assessment) and around 12 weeks postpartum.

### Clinical features of women with recurrence of depression

Clinical features, including information on psychiatric history and antidepressant use, were documented. SCID-I DSM-IV diagnoses, age of first and last onset of depression (SCID-I), number of depressive episodes (SCID-I), history of psychiatric hospital admission, psychiatric family history, antidepressant prescriber, current antidepressant dosage and number of previous discontinuation attempts were all determined around 16 weeks of pregnancy (baseline assessment).

The Edinburgh Postnatal Depression Scale (EPDS) was administered at baseline, 24 and 36 weeks of pregnancy, and 4 and 12 weeks postpartum for the assessment of depressive symptoms [28, 29]. The Beliefs about Medicine Questionnaire, specific version (BMQ-s) was administered at baseline [30]. The BMQ-s consists of two scales assessing 1) personal beliefs about the necessity of prescribed medication for controlling one’s illness (score range 5–25) and 2) concerns about the potential adverse consequences of taking medication (score range 6–30). Higher scores indicate stronger beliefs in the concepts of the scale. During follow-up, data on healthcare use was collected using the Trimbos/iMTA questionnaire for Costs associated with Psychiatric Illness (TIC-P) for number of visits to the general practitioner (GP), psychiatrist, psychologist, psychiatric nurse, psychotherapist and mental health care practice assistant [31].

### Vulnerability markers associated with recurrence of depression

Potential vulnerability markers associated with recurrence focused on sociodemographic characteristics, illness (history) and antidepressant specifications.

Included participant characteristics were age, level of education, having a paid job, parity and planned pregnancy (yes/no). Level of education was categorized in low (primary/secondary education) and higher education.

Included illness (history) determinants were EPDS score around 16 weeks of pregnancy, number of depressive episodes, number of axis I psychiatric co-morbidities (the sum of both previous and current diagnoses) as measured with the SCID-I and a history of psychiatric hospital admission.

Antidepressant specifications included duration of antidepressant use, number of previous tapering attempts, and dose equivalency in early pregnancy. Dose equivalency was calculated by dividing the prescribed dosage by standard initial dosages, which are: citalopram 20mg, escitalopram 10mg, fluoxetine 20mg, fluvoxamine 100mg, paroxetine 20mg, sertraline 50mg and venlafaxine 75mg. Standard dosages were based on American and Dutch pharmaceutical treatment guidelines [32, 33]. During prospective follow-up, tapering and discontinuation was reported and divided into three categories: 1) no tapering, 2) intention to taper, but did not completely discontinue antidepressants during follow-up and 3) participant completely discontinued antidepressants at any point during follow-up, whether or not this discontinuation afterwards persisted throughout follow-up.

### Statistical analysis

We performed a case-series study describing individual characteristics of participants. Additionally, exploratory univariable analyses were used to qualify associations between recurrence and vulnerability markers (previous section). We used logistic regression analyses with recurrence as the outcome variable and the vulnerability markers entered one at a time as independent variables. Given the explorative nature of the study the issue of multiple testing is not relevant in our view. All associations were expressed as odds ratios (OR) with 95% confidence intervals (95%CI). All statistical analyses were performed with SPSS, version 25.0.

## Results

**Fig 1:**
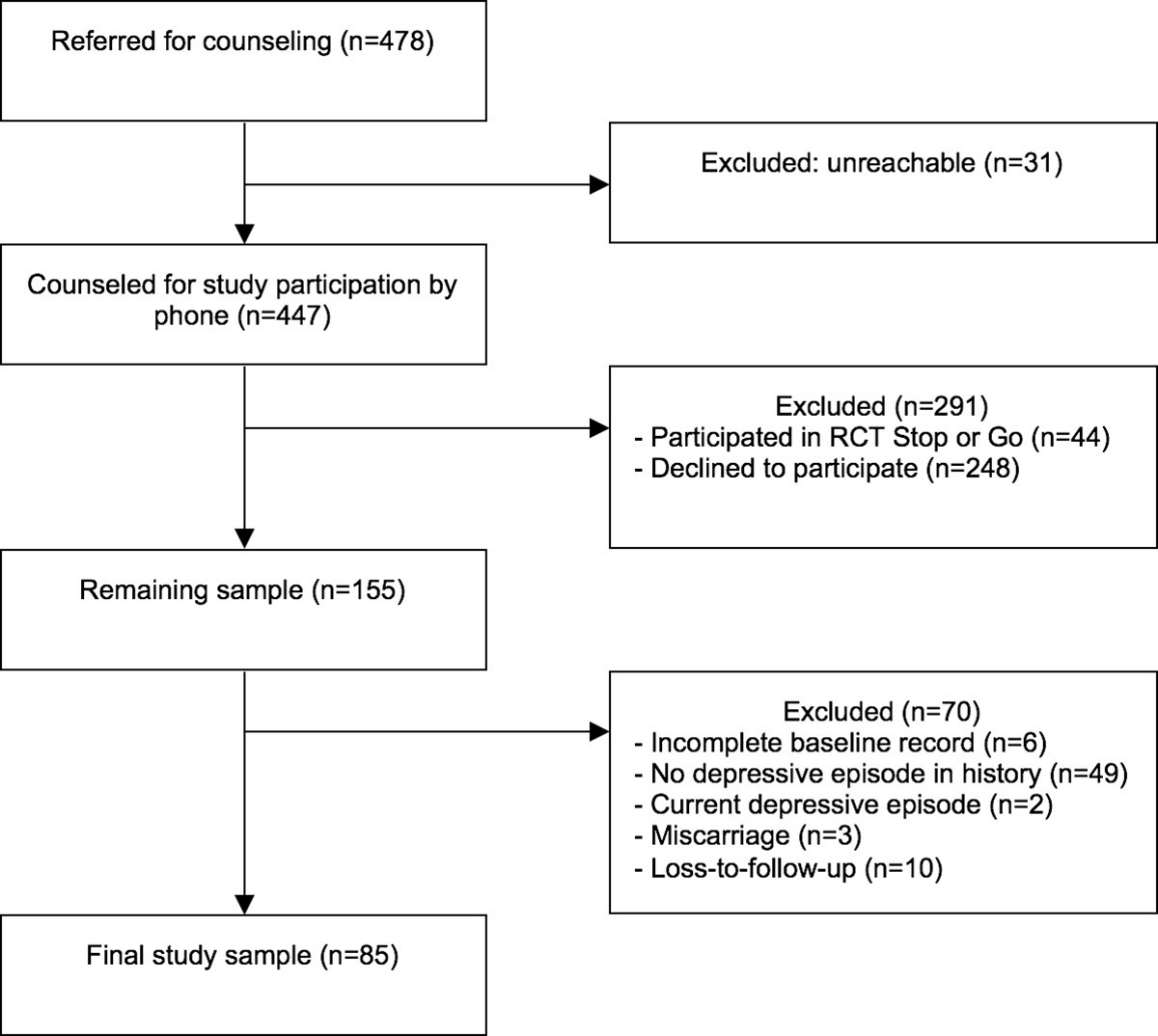
Flowchart of inclusion of participants.

A total of 478 pregnant women were referred for further counselling for both the RCT and the observational cohort. Thirty-one women (6.5%) were unreachable for counselling, 44 (9.2%) decided to participate in the RCT and 248 (51.9%) declined to participate in both trials. Of the remaining 155 women willing to participate in the observational cohort, another 70 (14.6%) were excluded for the current study: six had an incomplete baseline record, 49 did not have a history of depressive disorder, two were currently depressed, three had a miscarriage and ten were lost to follow-up. This resulted in a total sample of 85 women (Fig 1).

Table 1 illustrates the characteristics of all participants, including those with and without depression recurrence. Eight women (9.4%) experienced a perinatal recurrence of depression, none experienced relapse. Overall, mean age was 31.7 years (SD 4.1), 98.8% was of Dutch origin and 91.5% was living with her partner. Over half of the participants had a high level of education and for 50% it was their first pregnancy. Of all 85 women, 44 (51.8%) had one previous depressive episode and 7.1% had more than 3 episodes. Psychiatric co-morbidity was present in 71.8% of women (22.4% with current co-morbidity and 67.1% with previous co-morbidity), with panic disorder and agoraphobia most common (both 24.7% of participants). Overall, the median duration of antidepressant use was 60 months, during which a limited number of tapering attempts were undertaken; 81.0% either did not try or only once attempted to discontinue their antidepressants before study participation. During follow-up, 12 women (14.1%) completely discontinued their medication and four (4.7%) intended to taper medication, but did not completely discontinue during follow-up.

**Table 1:** Characteristics of pregnant women with a history of depression, with and without recurrence of depression during the perinatal period.

			All (n = 85)	Recurrence (n = 8)	No recurrence (n = 77)	OR (95% CI)
**Sociodemographic characteristics**						
	Age, mean (SD)		31.7 (4.1)	30.8 (5.1)	31.8 (4.0)	0.94 (0.78–1.12)
	High level of education, yes (%)		51 (65.4)	4 (57.1)	47 (66.2)	0.68 (0.14–3.29)
	Paid job, yes (%)		65 (79.3)	6 (75.0)	59 (79.7)	0.76 (0.14–4.17)
	Parity, median (range)		1.5 (1–11)	1 (1–5)	2 (1–11)	0.76 (0.38–1.54)
	Planned pregnancy, yes (%)		64 (78.0)	7 (87.5)	57 (77.0)	0.48 (0.06–4.17)
**Illness (history)**						
	EPDS score around 16 weeks of pregnancy, mean (SD)		6.7 (4.7)	11.6 (4.1)	6.2 (4.4)	1.28 (1.08–1.52)*
	No. of depressive episodes, median (range)		1 (1–10)	2.5 (1–6)	1 (1–10)	1.40 (0.94–2.07)
	No. of psychiatric co-morbidities, median (range)		1 (0–6)	2.5 (2–4)	1 (0–6)	1.89 (1.16–3.09)*
	History of admission to psychiatric institute, yes (%)		12 (14.1)	3 (37.5)	9 (11.7)	4.53 (0.92–22.26)
**Antidepressant specifications**						
	Duration of antidepressant use in months, median (range)		60.0 (4–252)	120 (12–228)	48 (4–252)	1.01 (1.00–1.02)*
	No. of tapering attempts in history, median (range)		1 (0–6)	0.5 (0–2)	1 (0–6)	0.65 (0.25–1.70)
	Dose equivalent at start study, mean (SD)		1.3 (0.6)	1.7 (0.7)	1.3 (0.6)	2.32 (0.84–6.42)
	Tapering antidepressants during follow up, n (%)		16 (18.8)	3 (37.5)	13 (16.9)	2.00 (0.89–4.53)
		Intention to taper, did not discontinue, n (%)	4 (4.7)	0 (0.0)	4 (5.2)	
		Discontinued during study, n (%)	12 (14.1)	3 (37.5)	9 (11.7)	

Columns may not sum due to missing data.

*p-value < 0.05

### Women with perinatal recurrence of depression

**Fig 2:**
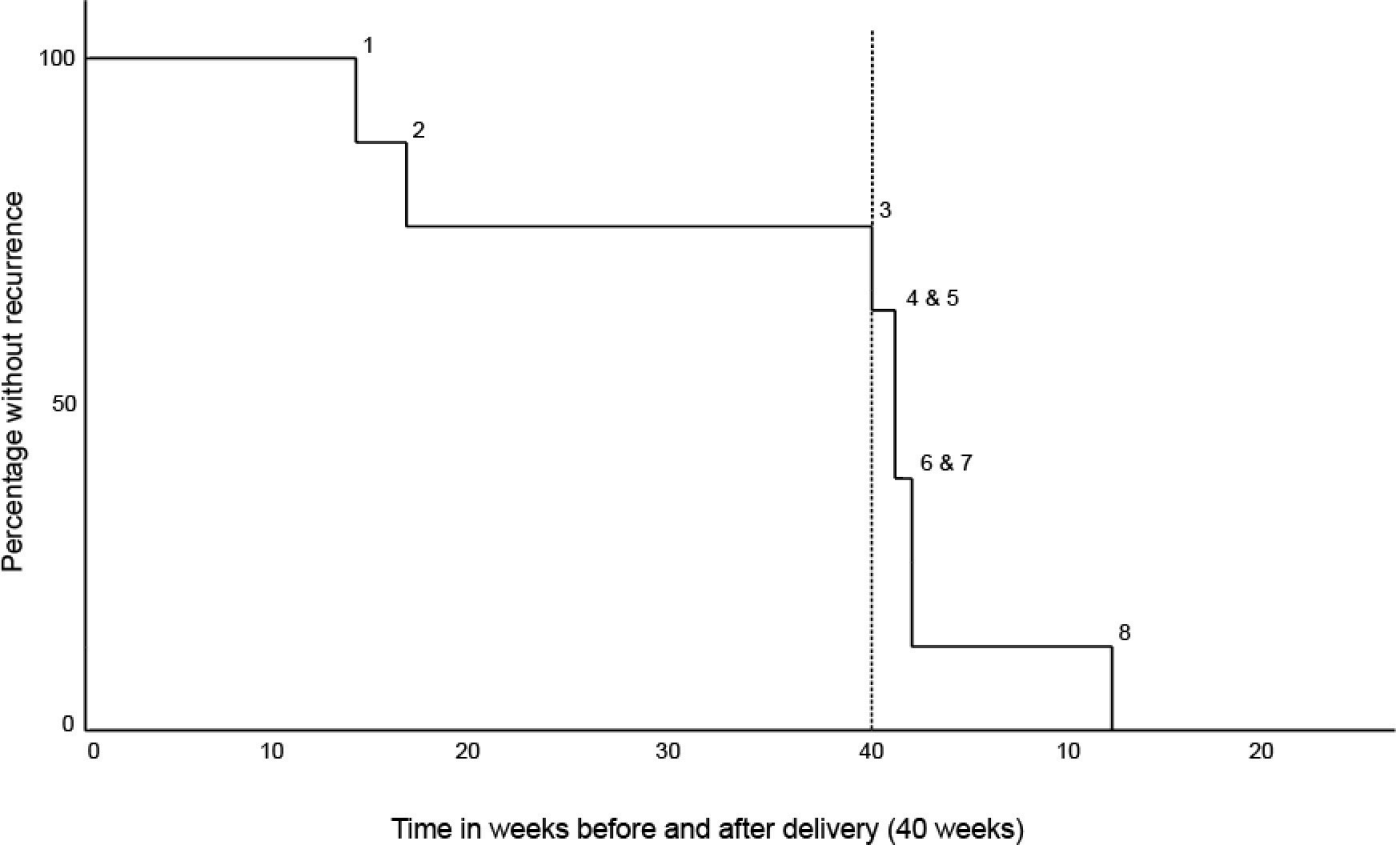
Survival curve of women with recurrence of depression in the perinatal period. Numbers in figure represent separate cases and match with case numbers listed in Table 2.

Out of eight women with a recurrence, six experienced recurrence after childbirth. Three of the women discontinued antidepressants during follow-up. A visual representation of timing of onset of recurrence can be seen in Fig 2. Clinical features of individual women are listed in Table 2 (case numbers correspond with case numbers in Fig 2). The mean age at first onset of depression was 16 years. None of these women had a previous episode with postpartum onset (for six women this was their first pregnancy). Four women had a positive family history of psychopathology; case 2 had a father with depression and obsessive-compulsive disorder, case 3 a brother with attention-deficit hyperactivity disorder and antidepressant use, case 7 a cousin who committed suicide and case 8 a mother, aunt and grandma with depression. All women had two or more psychiatric co-morbidities, mostly anxiety disorders. Most women received their medication through the general practitioner (GP). Overall, beliefs about necessity of their medication (BMQ-necessity) was high. However, the women with the lowest scores were also the women that discontinued their antidepressants during follow-up. The beliefs about adverse consequences were mild and homogeneous. All women had a history of (added) non-pharmacological treatment, receiving therapy for multiple years including cognitive therapy. Three women still received non-pharmacological therapy in early pregnancy.

**Table 2:** Clinical features of women with recurrence of depression in the perinatal period.

		Illness characteristics							Antidepressant specifications					Follow-up	
Case no.	Age	Parity	Age at first onset	Age at last onset	No. of episodes	Psych. co-morbidities	History of admission	Family history	Prescriber AD	BMQ nec/adv	Duration AD use	Baseline dose equiv	No. tapering attempts	Discon-tinued AD	No. psych care visits
**1**	31	1	14	14	1	2	-	-	GP	17/19	180	2.0	1	-	14
**2**	32	2	12	28	3	2	+	+	GP	23/21	120	3.0	1	-	18
**3**	37	5	17	28	3	3	-	+	GP	15/15	228	1.0	0	+	3
**4**	37	1	18	27	6	3	-	-	Psych	23/16	120	2.0	2	-	22
**5**	22	1	16	19	2	3	+	-	Psych	14/18	60	1.5	0	+	26
**6**	29	1	13	24	3	2	+	-	GP	21/17	96	1.0	0	-	15
**7**	32	1	20	27	2	2	-	+	GP	23/19	120	2.0	0	-	0
**8**	26	1	18	24	2	4	-	+	GP	13/18	12	1.0	1	+	15

No. = number, AD = antidepressants, BMQ = beliefs about medicines questionnaire (necessity (nec); score range 5–25, higher score indicates stronger belief in necessity, adverse (adv); score range 6–30, higher score indicates stronger belief in potential adverse consequences), (-) No/negative, (+) Yes/positive, GP = general practitioner, Psych = psychiatrist

All eight women visited the GP on average four times during study follow-up. Women had several psychiatric healthcare professionals. Five women visited a psychiatrist (mean number of visits 4.2), four a psychologist (mean number of visits 7.8), four a psychiatric nurse (mean number of visits 10.8), four a mental health care practice assistant (mean number of visits 2.0) and two a psychotherapist (mean number of visits 5). Case 7 only visited her GP (four times in total). The other women visited at least one more healthcare professional (range 1–5).

**Fig 3:**
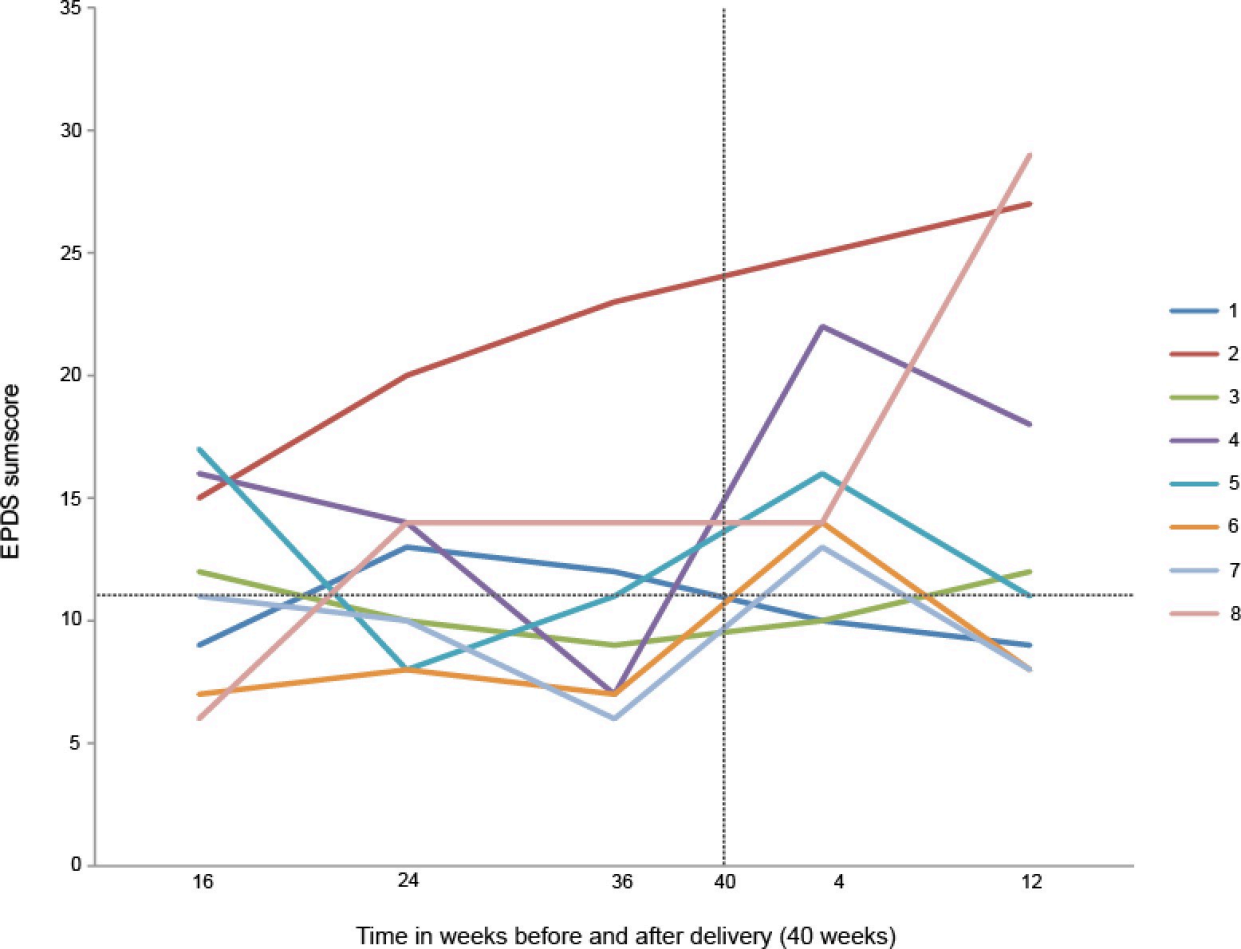
Edinburgh Perinatal Depression Scale (EPDS) sum score in the perinatal period. Each line represents a case with recurrence of depression. Case numbers match with case numbers listed in Table 2. The horizontal dotted line represents the EPDS cut-off score.

During pregnancy, the mean EPDS scores of the eight women remained stable (11.6, 12.1 and 11.1 consecutively), but increased after childbirth, around the time six women had a recurrence of depression. Mean EPDS scores around four weeks postpartum were 16.3, and 15.3 at 12 weeks postpartum. Fig 3 shows EPDS scores per case over time.

### Vulnerability markers associated with perinatal recurrence of depression

Univariable associations between the independent determinants and recurrence of depression are presented in Table 1. A higher EPDS score around 16 weeks of pregnancy (OR 1.28, 95%CI 1.08–1.52), a higher number of psychiatric co-morbidities (OR 1.89, 95%CI 1.16–3.09) and a longer duration of antidepressant use in months (OR 1.01, 95%CI 1.00–1.02) were associated with an increased risk of recurrence.

## Discussion

In this prospective cohort study, 85 pregnant women with a history of depression and baseline antidepressant use were assessed for depression recurrence. In total, eight women (9.4%) experienced a recurrence of depression at follow-up. All women with recurrence had experienced their first onset of depression during childhood/adolescence and had at least two psychiatric co-morbidities. Due to the low rate of recurrence, we were only able to explore univariable vulnerability markers associated with recurrence. Results yielded associations for recurrence with depressive symptoms around 16 weeks of pregnancy, number of psychiatric co-morbidities and duration of antidepressant use.

Two previous observational studies investigated recurrence rates of depression in women using antidepressant during pregnancy and reported rates ranging from 68 to 16% [23, 24]. Our overall recurrence rate (9.4%) is remarkably lower, despite the longer follow-up period. Of the women discontinuing, 25% experienced recurrence compared to 7% of the women continuing. A possible explanation may be the difference in study populations. Cohen et al. [23] included women through psychiatric institutes and reported that 76.6% had three or more previous depressive episodes. In the Yonkers study [24], 38% of the women had four or more previous depressive episodes, compared to 7.1% in our population. Number of previous episodes in the general population is one of the strongest predictors for recurrence [34]. Another explanation is that both previous studies reported most women had onset of recurrence in the first trimester [23, 24], whereas in our study, women with onset of recurrence in the first trimester (at start study) were excluded.

During follow-up, 14% of women completely discontinued their medication. This rate is in accordance with Dutch discontinuation rates during pregnancy as recorded in insurance and pharmacy databases [19, 35]. Similar to Yonkers et al. [24], our exploratory analysis did not find a significant effect of antidepressant discontinuation on recurrence risk. Outside of the perinatal period, antidepressants seem to be more protective than placebos in preventing recurrences, although this effect is not uniform [36] and recent trials have demonstrated tapering of antidepressants is safe when preventive cognitive therapy is provided [37, 38].

There were some noticeable details regarding the women with recurrent depression. All eight women had a first onset of depression during childhood or adolescence, and all but one of the women had multiple depressive episodes. Age of first onset has been associated with risk of recurrence, although it is difficult to disentangle the effect of age of first onset from the number of depressive episodes, as these two are highly correlated [39]. This early-onset depression has previously been associated with a more severe and chronic course of depression, often affecting women, with a longer duration of illness, more episodes, higher symptom severity, more psychiatric co-morbidity and more tendency to attempt suicide [40, 41].

Number of visits to psychiatric health care professionals during study follow-up ranged between 0 and 26 in all recurrence cases. Two women did not receive any, or only very limited, additional psychiatric healthcare, indicating that they did not receive adequate treatment for their recurrent depressive episode. This is unwanted as a review of 23 longitudinal studies found that 38% of mothers with postpartum depression (PPD) continued to have major depression during their child’s first year of life and even beyond, with previous history of depression as a predictor for a chronic course of PPD [42], affecting the child as well [13]. Ideally, all women would be offered additional care, even before recurrence takes place, as a recent study among adults showed that adding preventive cognitive therapy to antidepressant treatment resulted in a 41% relative risk reduction of relapse or recurrence of depression compared with antidepressants alone [37].

The exploratory analyses identified three vulnerability markers associated with recurrence: Depressive symptoms around 16 weeks of pregnancy, number of psychiatric co-morbidities and duration of antidepressant use. In early pregnancy, five women already had an EPDS score above cut-off [28], although they did not fulfil the SCID-I criteria yet for depressive disorder. The EPDS consists of ten questions and can thus be easily assessed in early pregnancy. International clinical guidelines encouraging routine screening for perinatal depression have been available for over a decade [43]. Previous validation research of the EPDS found that a cut-off value of 11 in the first trimester, and ten in the second and third trimesters gave the most adequate combination of sensitivity, specificity, and positive predictive value [28]. Clinicians may use these cut-off scores to initiate and monitor additional treatment, to prevent recurrence and decrease current symptoms of depression. The second marker was number of psychiatric co-morbidities. Psychiatric co-morbidity has been associated with shorter time to recurrence in a non-pregnant population [44]. During pregnancy, clinicians should therefore assess presence of psychiatric co-morbidities, and determine whether additional treatment targeting these co-morbidities is necessary. Lastly, a longer duration of antidepressant use was associated with recurrence. The OR must be interpreted as an increase of around 1% in the odds or recurrence per month. Longer antidepressant duration indicates maintenance treatment, which international guidelines recommend for patients with three or more depressive episodes [45].

### Strengths and limitations

A strength of the current study is that recruitment of participants took place in various settings (hospitals, midwifery practices, GP’s and social media) and detailed information was gathered prospectively, thereby preventing recall bias and providing insight into this specific population across the perinatal period. Moreover, clinical interviews were used to determine (history of) diagnoses throughout study participation instead of relying on self-report.

However, several limitations should be noted. Due to the inclusion and exclusion criteria, only women with recurrence of depression after the first trimester were included, limiting our sample size and thereby potentially the number of recurrences. With these limited numbers, only exploratory analyses could be conducted, and the possibility of confounding variables could not be ruled out. Results will have to be replicated in other studies with larger sample sizes. Other vulnerable groups of women, e.g. women with a history of depressive disorder who discontinue antidepressants before pregnancy, were not observed. In the Netherlands approximately 40% of women discontinue antidepressant treatment in the year before pregnancy and even higher figures are reported in other countries [19]. In the current study, women who discontinued antidepressants before pregnancy were excluded. To fully examine safety of antidepressant discontinuation, future studies should also include women discontinuing antidepressants before pregnancy. Yet, the main aim of the current study deliberately was to investigate the effects of continuation and discontinuation of antidepressants specifically during pregnancy. Another limitation is the limited number of women discontinuing their medication. To specifically examine the effect of discontinuation on recurrence rates, it would have been preferential to include more women discontinuing antidepressants, for example by oversampling this group. Lastly, we cannot guarantee generalizability of our results. Out of the 478 women referred for counselling, 248 women declined to participate, without providing a reason or background information, conforming to common and local ethical procedures. It remains unknown whether these women would have been eligible for participation and, if so, whether they would have differed significantly from our current study population. These women might have differed in their psychiatric history, current symptomatology and treatment management.

### Conclusion and future recommendations

The current study presented descriptive data on a prospective cohort of pregnant women with antidepressant use in early pregnancy and a history of depressive disorder. Three vulnerability markers associated with recurrence of depression were identified: Depressive symptoms in early pregnancy, number of current and past psychiatric co-morbidities and duration of antidepressant treatment. Importantly, if future studies can more robustly establish the predictive value of these vulnerability markers, these markers could easily be assessed as part of routine care procedures by clinicians. Implementing adequate and accessible risk assessment in daily practice can lead to improved individualized patient-centred care. No effect of discontinuation on risk of recurrence was observed, however, the proportion of women discontinuing medication was small and results should therefore be interpreted with caution. Future studies should aim to define additional predictors for recurrence of depression in pregnancy, assess the effects of implementing screening instruments during this phase, and to evaluate the effect of treatments in the perinatal period in order to benefit women and (potentially) their offspring [26, 46].
